# Echocardiographic Screening for Transcatheter Edge-to-Edge Mitral Valve Repair: Correlation Between Transthoracic and Transesophageal Assessment

**DOI:** 10.3390/jcdd12040149

**Published:** 2025-04-10

**Authors:** Michela Bonanni, Fausto Pizzino, Giovanni Benedetti, Rosangela Capasso, Rachele Manzo, Giuseppe Iuliano, Giancarlo Trimarchi, Andreina D’Agostino, Umberto Paradossi, Alessia Gimelli, Sergio Berti, Massimiliano Mariani

**Affiliations:** 1 Fondazione Toscana G. Monasterio, Ospedale del Cuore G. Pasquinucci, 54100 Massa, Italy; fpizzino@ftgm.it (F.P.); marianims@ftgm.it (M.M.); 2 Department of Experimental Medicine, University of Rome “Tor Vergata”, 00133 Rome, Italy; 3 Department of Clinical and Molecular Medicine, Division of Cardiology, Sapienza, University of Rome, 00185 Rome, Italy; capassorosangela@gmail.com; 4 Department of Advanced Biomedical Sciences, Federico II University of Naples, Via S. Pansini, 80131 Naples, Italy; rachele4manzo@gmail.com; 5 Cardiovascular Department, University Hospital “San Giovanni di Dio e Ruggi d’Aragona”, 84131 Salerno, Italy; gi.iuliano93@gmail.com; 6 Interdisciplinary Center for Health Sciences, Scuola Superiore Sant’Anna, 56127 Pisa, Italy; giancarlo.trimarchi18@gmail.com; 7 Department of Cardiac Imaging, Fondazione Toscana G. Monasterio, Via Giuseppe Moruzzi 1, 56124 Pisa, Italy

**Keywords:** transcatheter edge-to-edge repair, transthoracic echocardiography, transesophageal echocardiography, mitral regurgitation repair

## Abstract

Background: In patients with significant mitral regurgitation (MR) undergoing transcatheter edge-to-edge repair (M-TEER), assessment of mitral valve (MV) anatomy is essential. While transthoracic echocardiography (TTE) is the initial diagnostic tool, transesophageal echocardiography (TOE) provides better anatomical details. The study aims to assess whether TTE is as effective as TOE in selecting patients with severe MR who are eligible for M-TEER. Methods: From January to December 2024, patients with severe MR eligible for TEER were enrolled at the Fondazione Monasterio Heart Hospital, Italy. They underwent a comprehensive TTE and TOE examination. Cardiologists assessed the severity of MR and valve anatomy using specific protocols. Measurements included MV area, MV gradient, posterior leaflet length, fossa ovalis high, presence of fails, clefts, and calcifications. Three levels of anatomic complexity were defined to determine eligibility for TEER. Results: The study includes 40 patients with severe MR. The correlation between TTE and TOE for key parameters was strong, with coefficients ranging from 0.734 to 0.901, indicating high agreement between the two methods. The comparison of categorical features showed high agreement between TTE and TOE in detecting critical MV conditions, with kappa values ranging from 0.717 to 0.930. The agreement for classifying patients as suitable for M-TEER was 87.5%, indicating moderate consistency between the two methods. Conclusions: TTE may be a viable alternative to TOE for assessing MV anatomy and function before M-TEER in MR patients, especially in high-volume centers. While TTE strongly correlated with TOE for most parameters, TOE was superior for some features. Further research is needed to refine the clinical application of TTE and to define patient selection criteria for its use as the primary imaging modality for pre-procedural M-TEER screening.

## 1. Introduction

In patients with significant mitral regurgitation (MR) undergoing echocardiographic screening for transcatheter edge-to-edge repair (TEER), careful assessment of mitral valve (MV) anatomy is crucial to promote procedural success and minimize the risk of residual regurgitation or valve stenosis [1]. Transthoracic echocardiography (TTE) is the first diagnostic tool for evaluating MR mechanisms and severity, as well as for the assessment of other heart valve disease, biventricular function or the estimation of pulmonary artery pressures [2,3]. However, transesophageal echocardiography (TOE) is widely recognized as the best imaging technique for assessing MV anatomy and function before TEER due to its high spatial resolution and ability to provide a detailed, three-dimensional view of the valve [2,3,4]. In recent years, due to the increased number of procedures performed worldwide and the experience achieved, especially in high-volume centers, the extensive use of the EVEREST criteria for selecting patients with primary or secondary MR to TEER has become outdated [5,6,7,8,9,10,11]. Some of the parameters that determine anatomical complexity in MR include large coaptation gaps, complex flails, small MV area (MVA), high MV gradient, annulus or leaflet calcifications, multiple jets, leaflet–annulus index, and asymmetric tethering [10,11,12,13]. While degenerative MR often presents with highly variable and complex anatomical features that differ significantly from case to case, functional MR typically exhibits less anatomical complexity, often characterized by more consistent and predictable anatomical patterns, which can simplify procedural planning and decision making [7,8]. In this regard, we questioned if pre-procedural TOE is always necessary in specialized high-volume centers. The aim of our study is to evaluate whether in a selected subgroup of patients with MR, TTE may replace TOE in the morpho-functional assessment of MV and adequately screen patients for TEER.

## 2. Materials and Methods

### 2.1. Study Population

**Figure 1:**
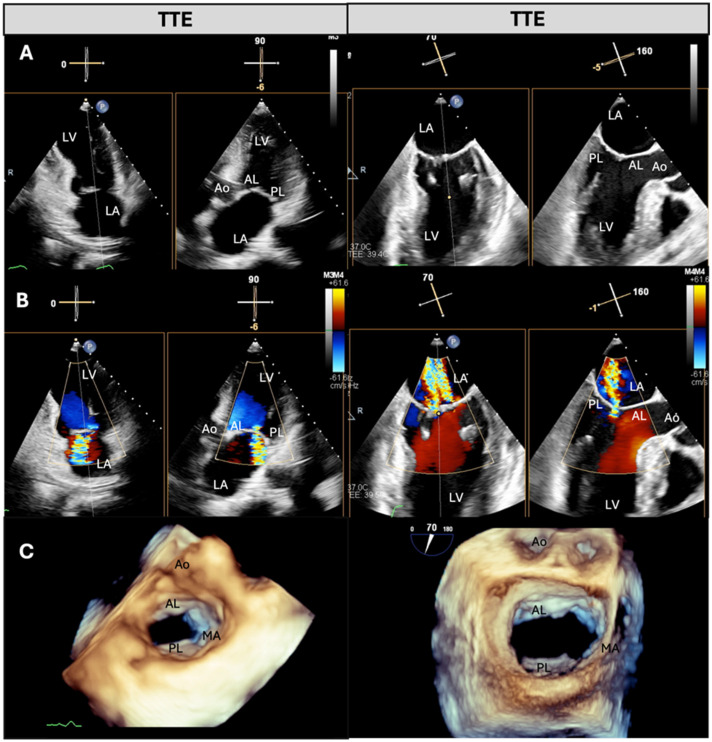
Comparison of TTE and TEE imaging in the assessment of functional mitral regurgitation. Example of functional mitral regurgitation assessed using transthoracic echocardiography (TTE, left) and transesophageal echocardiography (TEE, right). (**A**) B-mode images acquired using X-plane mode, showing the mitral valve structures with leaflet tethering due to left-ventricular dilation. (**B**) Color Doppler images were obtained with X-plane mode, showing the regurgitant jets extending into the left atrium. (**C**) Three-dimensional reconstructions of the mitral valve. The TEE 3D image provides higher anatomical resolution. Ao = aorta; AL = anterior leaflet; LA = left atrium; LV = left ventricle; MA = mitral annulus; PL = posterior leaflet.

**Figure 2:**
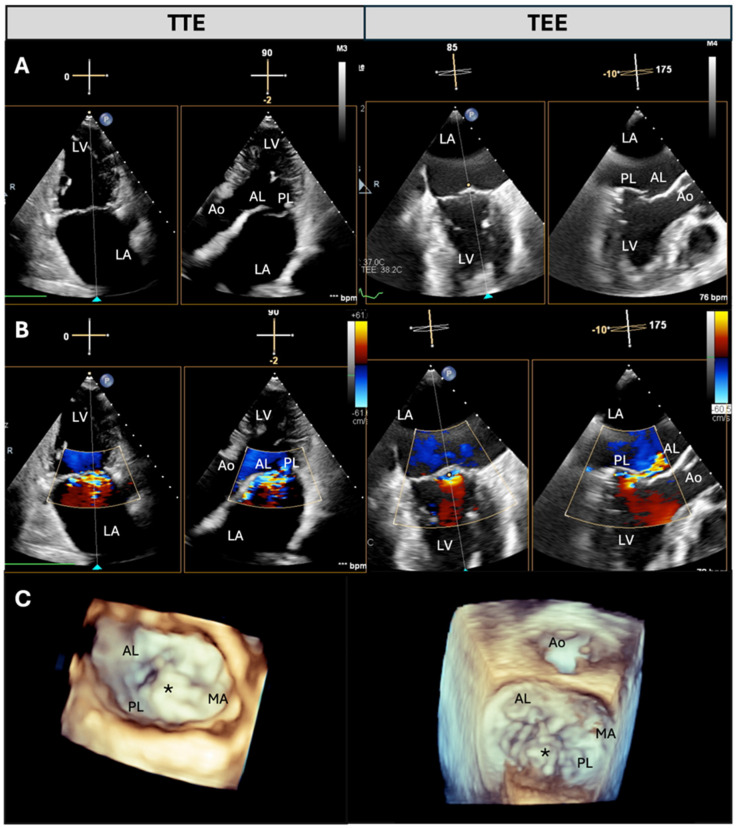
Comparison of TTE and TEE imaging in the assessment of degenerative mitral regurgitation with posterior leaflet flail. Example of degenerative mitral regurgitation with PL flail, assessed using transthoracic echocardiography (left) and transesophageal echocardiography (right). (**A**) B−mode images acquired using X−plane mode, showing PL flail. (**B**) Color Doppler images were obtained with X−plane mode, demonstrating severe mitral regurgitation with an eccentric jet. (**C**) 3D reconstructions of the mitral valve. The TEE 3D image clearly highlights the PL flail. * = flail segment; Ao = aorta; AL = anterior leaflet; LA = left atrium; LV = left ventricle; MA = mitral annulus; PL = posterior leaflet.

From January 2024 to December 2024, we prospectively enrolled patients with a diagnosis of severe MR who were eligible for TEER at the Fondazione Monasterio Heart Hospital, Massa, Italy. All patients underwent a comprehensive two-dimensional (2D) and three-dimensional (3D) TTE and TOE examination in our echo lab during the same visit [3,4,14]. Experienced cardiologists performed a specific protocol for MV assessment as part of a comprehensive TTE and TOE examination. All measurements were subsequently performed by two cardiologists, one dedicated exclusively to TTE and one dedicated to TOE. All patients provided written informed consent. For qualification purposes, the severity of MR was assessed according to the criteria recommended in the European guidelines for valvular heart disease [3]. This report compares measurements of the same parameters related to MR severity and mechanism performed with TTE and TOE (Figure 1 and [2]).

### 2.2. TTE Protocol

All TTE examinations were performed using a commercially available ultrasound system (Philips Epiq; Philips Medical Systems, Andover, MA, USA) equipped with the X5-1 probe. Multi-beat (when feasible) or single-beat 3D zoom imaging modality was used in the apical four-chamber view to assess the MV apparatus, including planimetric measurement of the MV area. The 3D images were cropped, rotated, and smoothed to visualize the MV from the atrial perspective (surgical view) [1,4]. The number of MR jets was assessed through 2D and 3D color Doppler. The quality of the images was evaluated based on the resolution of the MV and the presence or absence of artifacts throughout the cardiac cycle. Image quality was classified using a three-point scale as follows: score 1 = poor image quality (diagnostic possible, but with poor visibility of the anatomic details of the MV leaflet, difficult delineation of anatomic details); score 2 = good image quality (good visibility of the anatomical details of the MV); and score 3 = optimal (excellent visibility and differentiation of the anatomical details of the MV) (Figure 1 and Figure 2).

### 2.3. TOE Protocol

All TOE studies were performed using a commercially available ultrasound system (Philips Epiq; Philips Medical Systems, Andover, MA, USA) with a matrix array 3D TOE probe (X7-2 t; Philips Medical Systems). At the end of a comprehensive MV 2D examination, 3D images were acquired. For the 3D measurements, the MV was visualized in the mid-esophageal commissural view (approximately 50–70°), and 3D imaging of the MV was performed using the 3D zoom method, allowing for detailed and precise anatomical assessment [1,4,15] (Figure 1 and Figure 2).

### 2.4. Assessment of Anatomical Complexity for M-TEER Procedure

At the end of both TTE and TOE, the two cardiologists experienced in echocardiography provided a detailed assessment regarding the anatomical complexity of the M-TEER procedure. This evaluation was based on critical parameters for procedural success [10]. In our protocol, we analyzed and compared the following variables: (1) MV area (cm^2^); (2) MV gradient (mmHg); (3) posterior leaflet length (mm); (4) distance between fossa ovalis and MV leaflets coaptation point (mm); (5) presence of Barlow’s disease; (6) presence of flail leaflet; (7) evidence of bi-leaflet prolapse; (8) >2 independent significant jets; (9) presence of annulus and/or leaflet calcification, (10) presence of cleft. After these parameters were carefully analyzed, three levels of anatomical complexity related to the M-TEER procedure were identified: (1) non-complex valve anatomy, ideal for M-TEER; (2) complex valve anatomy, suboptimal for M-TEER but suitable in experienced centers; and (3) very complex valve anatomy, presenting significant challenges for M-TEER, even in highly experienced centers. The level of anatomic complexity was assessed independently by the two cardiologists, one evaluating the TTE assessment and the other evaluating the TOE assessment; these were reported separately.

## 3. Statistical Analysis

Continuous variables were expressed as mean ± standard deviation or median and inter-quartile range (IQR), according to Gaussian or non-Gaussian distribution. Normality was tested using the Shapiro–Wilks test. Categorical variables were expressed as frequencies and percentages. The different measurements were compared in the same patient using the paired Student *t*-test. The Pearson correlation coefficient was used to assess the relationship between TTE and TOE measurements. The agreement in categorical value between techniques was evaluated with Cohen’s Kappa value. All computations relied on commercially available software (SPSS IBMS v21 for Mac; SPPS Inc., Chicago, IL, USA and JMPw 11; SAS Institute Inc., Cary, NC, USA), with statistical significance set at *p* = 0.05.

## 4. Results

Clinical and echocardiographic characteristics of the study population are summarized in Table 1. Our study population consisted of 40 patients, including 15 (37.5%) women, with a mean age of 79 ± 6.7 years. MR etiology included 18 patients (45%) with degenerative MR, and 22 patients (30%) with functional MR. Of those with functional MR, 12 (30%) had ventricular etiology, and 10 (25%) had atrial functional MR. Regarding imaging quality, 9 cases (22.5%) were rated as optimal, 23 cases (57.5%) as sufficient, and 8 cases (20%) as insufficient for evaluating the feasibility of the M-TEER procedure. The mean effective regurgitant orifice area (EROA) was 0.36 ± 0.2 cm^2^, and the regurgitant volume (RV) averaged 55.9 ± 7.8 mL. The left-ventricular ejection fraction (LVEF) had a median value of 57%. The left-ventricular end-diastolic volume (LVEDV) was 100.5 mL, and the left-ventricular end-systolic volume (LVESV) was 42.5 mL. Procedural success, defined as a significant reduction in MR to grade ≤ 2+/4+ after device implantation, was achieved in 38 patients (95%).

### 4.1. Comparison of TOE and TTE Parameters Useful for Evaluating M-TEER Feasibility

**Figure 3:**
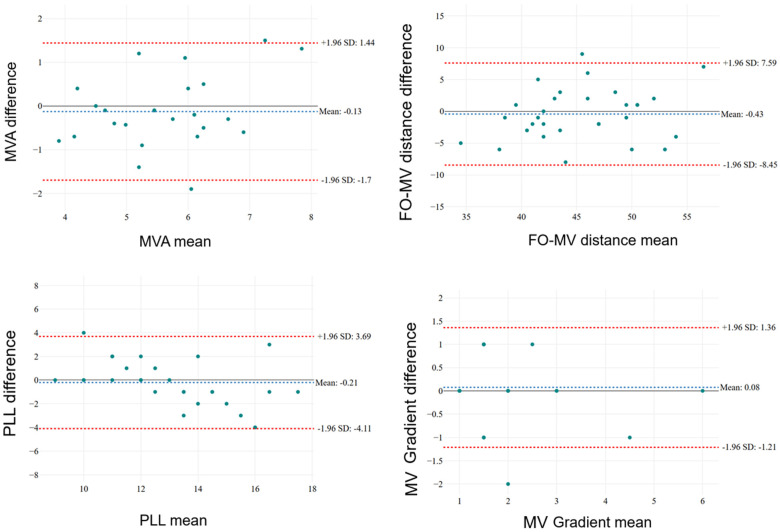
Bland–Altman plots. MVA: mitral valve area; FO: fossa ovalis, MV: mitral valve; PLL: posterior leaflet length.

Table 2 compares measurements obtained using TTE and TOE for several continuous variables. TOE showed higher mean values of MVA compared with TTE, but the difference was not statistically significant (*p* = 0.438). The mean transvalvular gradient was 1.9 ± 1.1 mmHg using TTE and 1.8 ± 1.3 mmHg with TOE (*p* = 0.714). The posterior leaflet length measured by TOE is slightly higher on average compared to TTE (13.5 mm vs. 13 mm), but the difference is not statistically significant (*p* = 0.641). Lastly, the height of the fossa ovalis was 44 ± 6.1 mm by TTE and 44.9 ± 5 mm by TOE, (IC = −1.970 to 1.355; *p* = 0.706). The Bland–Altman plot results further supports the idea that the two methods yield very similar results with minimal variation (Figure 3).

### 4.2. Correlation Between TOE and TTE Parameters in the Evaluation of M-TEER Feasibility

MVA and mean transvalvular gradient exhibit strong correlations, with coefficients of 0.750 (IC = 0.473–0.871) and 0.901 (IC = 0.799–0.952), respectively. The posterior leaflet length also has a high correlation coefficient (r = 0.734; IC = 0.503–0.867). Additionally, the fossa ovalis height demonstrates a strong correlation of 0.750 (IC = 0.524–0.871). Table 3 shows the agreement between TTE and TOE in assessing specific MV categorical features needed for evaluating M-TEER feasibility. For evidence of Barlow’s disease, TTE identified 5% of cases, and TOE identified 7.5%. The observed agreement between the two methods was 97.5%, with a kappa value of 0.787, (*p* < 0.001). In detecting flail, TTE reported 17.5% of cases, while TOE identified 27.5% with an observed agreement of 90%, and a kappa value of 0.717. Similarly, for bi-leaflet prolapse, TTE and TOE detected 5% and 7.5% of cases, respectively. The observed agreement was 95%, and the kappa value of 0.640 showed substantial agreement, with a *p*-value of <0.001 confirming statistical significance. For evidence of annular and/or leaflet calcification, both TTE and TOE identified 25% of cases. The observed agreement was 97.5%, and the kappa value of 0.930 suggested high agreement, (*p* < 0.001). In cases involving more than two independent jets, TTE detected 22.5% of cases, and TOE identified 32.5%. The observed agreement was 90%, and the kappa value of 0.752 indicated substantial agreement, again with statistical significance. Finally, for evidence of clefts, TTE did not identify any cases (0%), while TOE detected 7.5%. The observed agreement was 32.5%, suggesting very limited agreement between the two modalities for this feature. Regarding the technical feasibility of MR for M-TEER, the results showed substantial agreement between TTE and TOE (Table 3). For TTE, 68.8% of cases were categorized as “ideal” for M-TEER, 21.9% as “suboptimal”, and 9.4% as “challenging”. For TOE, 65.6% of cases were categorized as “ideal”, 28.1% as “suboptimal”, and 6.3% as “challenging”. The observed agreement between the two methods was 87.5%, and the Kappa value of 0.546 suggests moderate agreement. The result was statistically significant (*p* < 0.0001).

## 5. Discussion

The main findings of our study are as follows: (1) although TTE generally provided slightly lower values for MVA, posterior leaflet length and other important anatomical measurements, these differences were not statistically significant; (2) The two imaging modalities demonstrated moderate correlations in the assessment of MVA, transvalvular gradient, posterior leaflet length, and fossa ovalis height, indicating a reasonable but not complete concordance; (3) both TTE and TOE showed high agreement in identifying clinical anatomical features critical to M-TEER, such as annular and/or leaflet calcifications, and the presence of multiple independent jets; (4) There was a moderate agreement between TTE and TOE in categorizing patients based on anatomic complexity for M-TEER. Previous studies investigated the correlation between TTE and TOE methods in the assessment of MV. Grayburn et al. found a modest correlation between TTE and TOE in the assessment of functional MR and some anatomic parameters in patients with ischemic heart disease. Significant variability, likely due to differences in imaging planes and timing of measurements, limited the interchangeability of the methods [16]. Papadopoulos et al. investigated 3D assessment of the mitral annulus using TTE and TOE. Both methods showed strong correlations for the dimensions of the annulus, although some parameters, such as tenting height and intertrigonal distance, showed weaker agreement [17]. Berthelot-Richer M. et al. compared the measurement of mitral annulus area in degenerative MR using TTE and 3D-TOE. While the circular model of TTE using the apical 4-chamber view underestimated the area, 3D-TOE was found to be more accurate, emphasizing its superiority in accurately assessing the mitral annulus area [18]. Another study investigated the ability of 3D TTE to analyze the morphology of MV in degenerative MR. It showed high accuracy, good feasibility and improved sensitivity in identifying specific prolapse segments [19]. Additionally, a study comparing 3D TTE and 3D TOE in the preoperative evaluation of severe PMR found both methods equally effective in identifying diseased scallops, though 3D TTE was less reliable for detecting multiple chordal ruptures. Quantitative analysis showed consistent results for static annular parameters but differences in dynamic measurements. With dedicated 3D software, 3D TTE offers a thorough, non-invasive preoperative assessment, especially for static parameters [20]. To date, no study has specifically examined the use of TTE as the primary modality for assessing MV anatomy in patients scheduled for M-TEER. Traditionally, TOE has been the gold standard for assessing MV anatomy because it offers better image resolution and provides detailed views of the MV and surrounding structures. Our findings suggest that TTE may be a reliable alternative to TOE for evaluating MV anatomy in specific patient groups undergoing TEER. Given that TTE is less invasive, more accessible, and easier to perform than TOE, its use in high-volume centers could streamline the assessment, particularly in patients with less complex MV anatomy. Unlike TOE, which often requires sedation and specialized expertise, TTE can be performed at the bedside and provides immediate results without the need for additional resources. In patients with uncomplicated MR or simple MV anatomy, TTE can provide sufficient imaging data for accurate pre-procedural assessment, potentially reducing the need for more invasive imaging procedures. In our study, in most patients, TTE and TOE provided comparable information on key parameters. However, in eight patients, TTE was deemed insufficient for reliably assessing the feasibility of M-TEER. This limitation underscores the challenges of using TTE as a stand-alone imaging modality in cases of anatomical complexity or poor image quality. Notably, five of these cases involved degenerative MR, which is often associated with more complex and variable valve anatomy, such as valve prolapse, clefts, or flail leaflets. This suggests that while TTE can accurately assess most parameters required for M-TEER in the majority of patients, TOE may still offer an advantage in evaluating MV with challenging anatomical features. This finding emphasizes the importance of tailoring imaging modality choice to the patient’s specific MV pathology, with TTE being particularly useful for preoperative screening in functional MR, where valve anatomy is typically less complex.

### Limitations

Firstly, the sample size of 40 patients is relatively small, which could limit the generalizability of the results. Second, the study only included patients from a single high-volume center, meaning that the results might not be generalizable to lower volume centers with less experience with TEER procedures. Third, the subjective nature of image quality assessment in both TTE and TOE may also lead to variability in the interpretation of results despite the use of a standardized three-point scale. Lastly, although no statistically significant differences were observed between the measurements obtained with TTE and TOE, this should not be misinterpreted as an indication of statistical equivalence between the two modalities. Instead, it suggests the absence of a systematic measurement bias in TTE. However, as highlighted by the correlation coefficients and Bland–Altman plots, substantial variability exists at the individual patient level. This implies that while systematic error appears negligible, random error may still affect the reliability of TTE in specific cases. Consequently, careful patient selection and further validation studies are necessary to determine the true clinical applicability of TTE as a standalone imaging modality for TEER qualification.

## 6. Conclusions

TTE represents a promising alternative to TOE for the preprocedural assessment of MV anatomy and function in patients with MR, particularly in specialized high-volume centers. While TTE demonstrated a moderate correlation with TOE across most anatomical parameters, TOE still offered superior performance in certain aspects. Given TTE’s advantages of greater accessibility, ease of use, and non-invasive nature, further studies are needed to refine its clinical application and to identify optimal patient selection criteria for its use as the primary imaging modality in M-TEER.

## Figures and Tables

**Table 1:** Clinical and echocardiographic characteristics.

Variables	Total (N = 40)
Age, yrs	79 ± 6.7
Female, N (%)	15 (37.5)
Atrial fibrillation, N (%)	25 (62.5)
Previous HF hospitalization, N (%)	12 (30)
CRT-D, N (%)	2 (5)
ICD, N (%)	2 (5)
Previous MI, N (%)	16 (40)
B-blockers, N (%)	26 (65)
ACE-I/ARB/ARNI, N (%)	30 (75)
MRA, N (%)	20 (50)
Furosemide > 50 mg, N (%)	15 (37.5)
SGLT2i, N (%)	15 (23.4)
NYHA ≥ III, N (%)	29 (72.5)
egenerative MR, N (%)	18 (45)
Functional MR, N (%)	
- ventricular, N (%) - atrial, N (%)	12 (30) 10 (25)
Imaging quality	
- Insufficient - Sufficient - Optimal	8 (20) 23 (57.5) 9 (22.5)
EROA, cmq	0.36 ± 0.2
RV, mL	55.9 ± 7.8
LVEF, %	57 (46.25–66.74)
LVEDD, mm	52 (48–58)
LVESD, mm	33 (27.5–40)
LVEDV, mL	100.5 (80–126)
LVESV, mL	42.5 (25.25–66)
LA area, cmq	26 (21–31.5)
LA volume, mL	86 (68–129)
PAPs, mmHg	43 (33–60)
TR (≥moderate), N (%)	5 (12.5)

ACE-I: angiotensin-converting enzyme inhibitor; ARB: angiotensin receptor blocker; ARNI: angiotensin receptor-neprilysin inhibitor; CRT-D: cardiac resynchronization therapy with defibrillator; EROA: effective regurgitant orifice area; HF: heart failure; ICD: implantable cardioverter-defibrillator; LA: left atrium; LVEDD: left-ventricular end-diastolic diameter; LVEDV: left-ventricular end-diastolic volume; LVEF: left-ventricular ejection fraction; LVESD: left-ventricular end-systolic diameter; LVESV: left-ventricular end-systolic volume; MI: myocardial infarction; MR: mitral regurgitation, MRA: mineralocorticoid receptor antagonist; NYHA: New York heart association functional class; PAPs: pulmonary artery systolic pressure; RV: regurgitant volume; SGLT2i: sodium-glucose cotransporter-2 inhibitor; TR: tricuspid regurgitation.

**Table 2:** Comparison of TOE and TTE measurements for evaluating M-TEER feasibility.

Variables	TTE Mean ± SD	TOE Mean ± SD	95% CI	Paired *t*-Test	Bland–Altman
MVA, cm^2^	5.5 ± 1.1	5.8 ± 1.2	−0.466–0.208	0.438	0.323
Transvalvular gradient, mmHg	1.9 ± 1.1	1.8 ± 1.3	−1.970–1.3559	0.714	0.109
Posterior leaflet length, mm	13 ± 2	13.5 ± 3.3	−1.032–0.047	0.641	0.000
Distance septal puncture point-mitral valve	44 ± 6.1	44.9 ± 5	−1.970–1.355	0.706	0.635

CI: confidence interval; MVA: mitral valve area; TTE: transthoracic echocardiography; TOE: transesophageal echocardiography.

**Table 3:** Correlation between TOE and TTE in the evaluation of categorical parameters useful for assessing M-TEER feasibility.

Variables	TTE N (%)	TOE N (%)	Observed Agreement (%)	Kappa Value	*p*-Value
Evidence of Barlow’s disease	2 (5)	3 (7.5)	97.5	0.787	<0.001
Evidence of flail	7 (17.5)	11 (27.5)	90	0.717	<0.001
Bi-leaflet prolapse	2 (5)	3 (7.5)	95	0.640	<0.001
Evidence of annular and/or leaflet calcification	10 (25)	10 (25)	97.5	0.930	<0.001
>2 independent jets	9 (22.5)	13 (32.5)	90	0.752	<0.001
Evidence of cleft	0 (0)	3 (7.5)	32.5		
Technical feasibility of MR *					
- Ideal for M-TEER - Suboptimal for M-TEER - Challenging for M-TEER	22 (68.8) 7 (21.9) 3 (9.4)	21(65.6) 9 (28.1) 2 (6.3)	87.5	0.546	<0.000

* Patients for whom the feasibility of the M-TEER procedure could not be assessed using TTE are excluded from this calculation. MR: mitral regurgitation; M-TEER: mitral-transcatheter edge-to-edge repair; TTE: transthoracic echocardiography; TOE: transesophageal echocardiography.

## Data Availability

Data are available from the corresponding authors under reasonable request.
